# eHealth Implementation Issues in Low-Resource Countries: Model, Survey, and Analysis of User Experience

**DOI:** 10.2196/23715

**Published:** 2021-06-18

**Authors:** Norman Archer, Cynthia Lokker, Maryam Ghasemaghaei, Deborah DiLiberto

**Affiliations:** 1 Information Systems DeGroote School of Business McMaster University Hamilton, ON Canada; 2 Health Research Methods, Evidence and Impact Faculty of Health Sciences McMaster University Hamilton, ON Canada; 3 Global Health Faculty of Health Sciences McMaster University Hamilton, ON Canada

**Keywords:** eHealth, low-resource countries, eHealth implementation effectiveness, end user survey, eHealth utilization

## Abstract

**Background:**

The implementation of eHealth in low-resource countries (LRCs) is challenged by limited resources and infrastructure, lack of focus on eHealth agendas, ethical and legal considerations, lack of common system interoperability standards, unreliable power, and shortage of trained workers.

**Objective:**

The aim of this study is to describe and study the current situation of eHealth implementation in a small number of LRCs from the perspectives of their professional eHealth users.

**Methods:**

We developed a structural equation model that reflects the opinions of professional eHealth users who work on LRC health care front lines. We recruited country coordinators from 4 LRCs to help recruit survey participants: India, Egypt, Nigeria, and Kenya. Through a web-based survey that focused on barriers to eHealth implementation, we surveyed 114 participants. We analyzed the information using a structural equation model to determine the relationships among the constructs in the model, including the dependent variable, eHealth utilization.

**Results:**

Although all the model constructs were important to participants, some constructs, such as user characteristics, perceived privacy, and perceived security, did not play a significant role in eHealth utilization. However, the constructs related to technology infrastructure tended to reduce the impact of concerns and uncertainties (path coefficient=−0.32; *P*=.001), which had a negative impact on eHealth utilization (path coefficient=−0.24; *P*=.01). Constructs that were positively related to eHealth utilization were implementation effectiveness (path coefficient=0.45; *P*<.001), the countries where participants worked (path coefficient=0.29; *P*=.004), and whether they worked for privately or publicly funded institutions (path coefficient=0.18; *P*<.001). As exploratory research, the model had a moderately good fit for eHealth utilization (adjusted R^2^=0.42).

**Conclusions:**

eHealth success factors can be categorized into 5 groups; our study focused on frontline eHealth workers’ opinions concerning 2 of these groups: technology and its support infrastructure and user acceptance. We found significant disparities among the responses from different participant groups. Privately funded organizations tended to be further ahead with eHealth utilization than those that were publicly funded. Moreover, participant comments identified the need for more use of telemedicine in remote and rural regions in these countries. An understanding of these differences can help regions or countries that are lagging in the implementation and use of eHealth technologies. Our approach could also be applied to detailed studies of the other 3 categories of success factors: short- and long-term funding, organizational factors, and political or legislative aspects.

## Introduction

### Background

eHealth is the cost-effective and secure use of information communication technology in support of health and health-related fields, including health-care services, health surveillance, health literature, and health education, knowledge and research [1].

Properly implemented eHealth has the potential to scale up the delivery of health care to people in low-resource countries (LRCs) [2]. Quality of health care has been found to contribute the most to the success of these eHealth interventions, whereas cost contributes the most to intervention failures [3]. The challenges faced when implementing eHealth vary among countries. For eHealth solutions to succeed in LRCs, an organized approach must be used to address these challenges.

A 2016 World Health Organization report [1] indicates significant progress in eHealth planning:

More than half of WHO Member States now have an eHealth strategy, and 90% of eHealth strategies reference the objectives of UHC [Universal Health Coverage] or its key elements. It is becoming mainstream for countries to have policies for managing information. When well-articulated, eHealth strategies should enable the interoperability needed to support people-centred health services for everyone, and the move from disease silos to resilient health systems which can deliver UHC.

Although the World Health Organization claims that more than half of their member states have an eHealth strategy, actual implementation of their strategies is not always followed. This is clear from published studies and concerns expressed by many researchers [3-7]. For example, Kiberu et al [4] suggest that although many sub-Saharan African countries are evaluating eHealth as a means of improving health care accessibility, several are engaged in the proof-of-concept stage of unsustainable pilot projects. There are no national guidelines in many LRCs for the secure management of individual digital health information and services, placing personal data at risk. Implementation issues with standards and interoperability can create barriers to the use of eHealth and its spread across regions or nations to support the full realization of potential health system benefits. The potential for eHealth to reduce health care costs and enable access to better quality health care is limited, often due to inadequate funding, inadequate infrastructure causing power blackouts, poor internet connectivity, and an unskilled eHealth workforce. However, regardless of having to work in such difficult circumstances, progress is being made in implementing eHealth in many LRCs. For example, a few of the many research papers that have been published by LRC researchers include critical issues such as eHealth being used to combat infant mortality in rural and remote regions of India [8] and Nigeria [9], open-source electronic health record systems that support interoperable links among them have been installed in Kenya [10], and telecom policies developed to encourage optimal digital network implementation to support eHealth in Egypt [11].

### Objective

The objective of this study is to create and validate a model of the factors that influence the successful implementation of eHealth in LRCs, based on eHealth challenges identified in a survey of LRC eHealth end users. A recent categorization [12], modified by Ahmed et al [13], has synthesized eHealth success factors into 5 categories: (1) technology and its support infrastructure, (2) user acceptance, (3) short- and long-term funding, (4) organizational factors, and (5) political or legislative aspects. Our study focuses on factors 1 and 2, which are likely to be of interest and intimately familiar to the end user participants we surveyed.

## Methods

### Construct Development for the eHealth Implementation Issues Model

**Figure 1:**
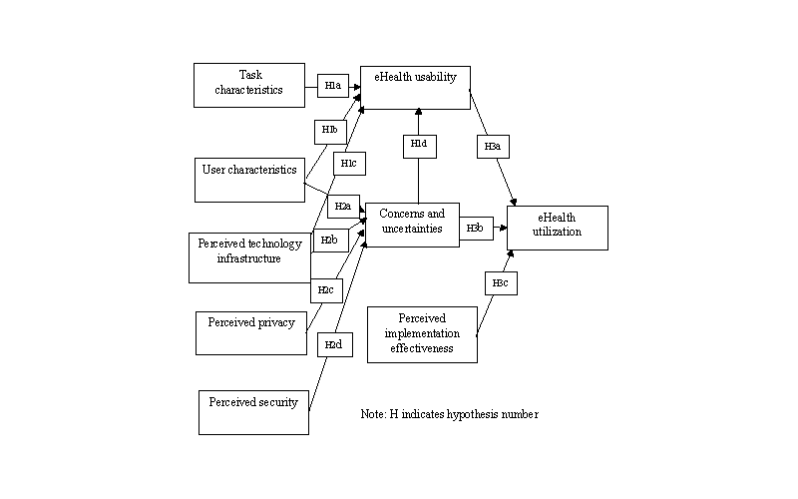
Structural equation model of eHealth implementation issues in low-resource countries.

Figure 1 shows the model used in this study. The following discussion describes the details of the model’s development and related hypotheses (shown in small rectangles in the diagram), including the background of the model constructs. The study based on this model was implemented through a web-based questionnaire detailed in Multimedia Appendix 1.

#### Task Characteristics

Tasks are the activities that support the organization. Information systems facilitate completion of the organization’s tasks. Compatibility with work processes or work styles and task difficulty are often used to characterize tasks [14]. Research has shown [15] that if a system is more aligned with the needs of the users, there is a greater chance of system success. A fit-variability model [16] (related to the task-technology fit model by Goodhue [15]) showed that different stakeholders may perceive eHealth viability and fit of the same eHealth services very differently and that there can be discrepancies between organizational viability and individual fit of specific eHealth services. Relevant information from the study by Goodhue and Thompson [15] was used to derive the task characteristics construct for the questionnaire (Multimedia Appendix 1). This leads to:

- Hypothesis 1a: Task characteristics will positively affect eHealth usability.

#### User Characteristics

“User characteristics are attitudes, perceptions, and demographics that are specific to the individual users of the information system” [14]. A survey of 465 medical professionals in northwest Nigerian hospitals [7] showed that the majority of the participants had a good level of literacy for implementing and working with new eHealth systems. They found statistically significant positive correlations between intention to use eHealth and attitude toward eHealth, perceived usefulness toward eHealth, information technology experience and eHealth, and technical infrastructure for eHealth. The user characteristics construct for the questionnaire (Multimedia Appendix 1) was adapted from the study by Zayyad and Toycan [7]. This leads to:

- Hypothesis 1b: User characteristics will positively affect eHealth usability.
- Hypothesis 2a: User characteristics will negatively affect concerns and uncertainties.

#### Perceived Technology Infrastructure

Information communications and technology is a key component of any eHealth program. For an eHealth program to succeed, technology must be available to support a database that is always directly and easily accessible to practitioners for health record input, retrieval, analysis, and sharing within the patient’s circle of care. The infrastructure must include stable and reliable digital communications network hardware and software to support health record access and sharing through point-of-care devices used by health care providers. Highly reliable electrical power must also be available at all times to drive the components of the infrastructure [4,6,7,17]. The relevant perceived technology infrastructure construct for the questionnaire (Multimedia Appendix 1) was adapted from the study by Zayyad and Toycan [7] as follows:

- Hypothesis 1c: Perceived technology infrastructure will positively affect eHealth usability.
- Hypothesis 2b: Perceived technology infrastructure will negatively affect concerns and uncertainties.

#### Perceived Privacy

A study of eHealth in Bangladesh found that privacy concerns by patients [18] did not have a significant impact on provider concerns about eHealth use. An explanation is that poor eHealth privacy and security considerations resulted in eHealth systems being judged by patients as failures [19]. This is the opposite of the findings from users of eHealth (clinicians) who may tend to believe that privacy and security are less important when an eHealth system provides superior health care. The perceived privacy construct for the questionnaire (Multimedia Appendix 1) was derived from the study by Wilkowska and Ziefle [20], as follows:

- Hypothesis 2c: Perceived privacy will positively affect concerns and uncertainties.

#### Perceived Security

Security and privacy issues are related in eHealth systems; it is not possible to manage privacy without a secure system. A survey of professionals working in Nigerian hospitals addressed the professionals’ intentions to use eHealth [7]. A question related to security concerns showed a nonsignificant correlation of this issue with the intention to use eHealth. The perceived security construct of the questionnaire (Multimedia Appendix 1) was derived from the study by Wilkowska and Ziefle [20] as follows:

- Hypothesis 2d: Perceived security will positively affect concerns and uncertainties.

#### eHealth Usability

Usability represents an important yet often overlooked factor impacting the implementation and meaningful use of eHealth systems. Without usable systems, doctors, medical technicians, nurses, administrative staff, and other users would have great difficulty in realizing the potential benefits of eHealth systems. The usability of technical systems has been studied in the information systems field, beginning with the landmark work by Davis [21]. This is a key measure of an eHealth system and directly reflects how users may react positively to its use. In general, if the system is built to perform specific user tasks, its usability will be greater. The eHealth usability construct for the questionnaire (Multimedia Appendix 1) was adapted from the study by Davis [21]. The foregoing leads to the following:

- Hypothesis 3a: eHealth usability will positively affect eHealth utilization.

#### Concerns and Uncertainties About eHealth

A review of empirical research classifying eHealth implementations as successes or failures [3] found that quality of health care was most often mentioned as contributing to the success of eHealth interventions. This review found that costs were most frequently mentioned as contributing to failure, although workflow issues were also mentioned in most of the articles reviewed. Workflow issues could lead to disagreement among the affected clinicians, increasing uncertainties, and the potential for failure of the eHealth system. The concerns and uncertainties about eHealth construct of the questionnaire (Multimedia Appendix 1) adapted ideas expressed by Aranda-Jan et al [22] in a systematic review of what does not work in African eHealth projects, leading to the following hypotheses:

- Hypothesis 1d: Concerns and uncertainties about eHealth systems will negatively affect eHealth usability.
- Hypothesis 3b: Concerns and uncertainties about eHealth systems will negatively affect eHealth utilization.

#### Perceived Implementation Effectiveness

Underlying factors affect health care professionals’ decisions to implement eHealth technology applications in LRCs [7]. These include the perceived usefulness, belief, willingness, and attitude of health care professionals. Our study implicitly reflects these factors in terms of survey feedback from users who have chosen to implement eHealth in their workplaces. The technological capability of eHealth systems is one of the key factors that influence the successful implementation of a technology [17]. Technological success factors include functional and nonfunctional requirements, interoperability, and user interface design. The long-term sustainability of a system depends on the economic, social, and organizational sustainability in which the technology is embedded. The perceived eHealth implementation effectiveness construct of the questionnaire (Multimedia Appendix 1) was developed from ideas expressed by Rezai-Rad et al [23] and is stated as:

- Hypothesis 3c: Perceived implementation effectiveness will positively affect eHealth utilization.

#### eHealth System Utilization

Utilization of an eHealth system is a measure of how popular the system is with the users and if it will be sustainable and worth the operating cost in the long run. This was measured in our study by eHealth system utilization, a one-indicator formative construct Q10 (Multimedia Appendix 1) that lists possible eHealth utilization levels by the participant’s organization.

Table 1 summarizes the reference sources mentioned above that were used to create the eHealth implementation model.

**Table 1:** Summary of sources for eHealth implementation model constructs.

Title	Construct	Type	Study
Perceived task characteristics	Validated	Reflective	Goodhue and Thompson [15]
Individual characteristics	New	Reflective	Zayyad and Toycan [7]
Perceived technology infrastructure	New	Reflective	Zayyad and Toycan [7]
Perceived eHealth privacy	Validated	Reflective	Wilkowska and Ziefle [20]
Perceived eHealth security	Validated	Reflective	Wilkowska and Ziefle [20]
eHealth usability	Validated	Reflective	Davis [21]
Concerns and uncertainties about eHealth	New	Reflective	Aranda-Jan et al [22]
eHealth implementation effectiveness	New	Reflective	Rezai-Rad et al [23]
eHealth utilization	New	Formative (1-indicator variable)	N/A^a^

^a^N/A: not applicable (as this construct is developed in this study).

### Implementation

#### Overview

This study was approved by the McMaster University Research Ethics Board. In addition to its own approval process, the board contacted eHealth authorities in each of the 4 LRCs to ensure that the research proposal was acceptable. The study proceeded in 2 phases: I and II. An individual country coordinator with experience in eHealth implementations was recruited for each of the 4 countries involved in the study. They received nominal reimbursement for managing the recruitment of eHealth expert consultants for phase I and survey participants for phase II in their respective countries. eHealth consultants in the phase 1 study also received a nominal sum. Participants were not paid for completing the survey (phase II). In the 2 countries where 35 or more participants were recruited, a random draw prize was awarded to 1 participant in each country.

#### Phase I

On the basis of a detailed review of the relevant eHealth literature, we developed a draft questionnaire. Our study focused on eHealth users and support staff in the 4 representative LRCs: Kenya (East Africa), Nigeria (West Africa), India (South Asia), and Egypt (North Africa). Consultations with the experts on the phase I questionnaire informed the design of the final questionnaire and highlighted the importance of the factors identified from the literature. A key finding was that the model was too broad, including a number of strategic concerns that individual participants, as clinicians or other end users, were less likely to be directly concerned with. These strategic concerns (3 of the 5 categories [12] referenced earlier in this paper: short- and long-term funding, organizational factors, and political or legislative aspects) made the questionnaire too long for busy eHealth users to be willing to complete. Therefore, we reduced the scope of the issues covered to the first 2 categories (technology and its support infrastructure and user acceptance). The final questionnaire was based on the more limited model shown in Figure 1. Each reflective construct in the model included at least three indicator variables, which were presented on a 7-point Likert scale. The resulting questionnaire is provided in Multimedia Appendix 1, and the information, consent, and invitation to participate messages to the survey participants are provided in Multimedia Appendix 2. The web-based version, developed using *Qualtrics* software[24], took about 15 minutes for the participants to complete.

#### Phase II

A convenience sampling survey of eHealth users in the 4 LRCs was arranged by the relevant country coordinators, who recruited suitable participants. Participants were from public and private institutions in rural and urban areas and had varying levels of eHealth experience. Details of the survey process are provided in Multimedia Appendix 3. From 177 invitations to participate, 114 (64.4% overall response rate) valid responses were completed from India (39/114, 34.2%), Egypt (52/114, 45.6%), Kenya (11/114, 9.6%), and Nigeria (12/114, 10.5%). Statistical data were analyzed using partial least squares with *Smart PLS3* software [25].

## Results

### Participant Demographics

Participant demographics are detailed in Table 2, including country comparisons. In the table, the *Total* column represents the number of participants from each country who completed the survey successfully. All data are presented as absolute values and percentages. Percentages in the total column on the righthand side sum to 100% for the categories presented in each of the 5 table divisions that were also used, along with country, as control variables (occupation, employer, eHealth experience, urban or rural experience, and employment experience).

**Table 2:** Participant demographics.

Characteristics			Country, n (%)									Total (N=114), n (%)
			India (n=39)		Egypt (n=52)		Kenya (n=11)		Nigeria (n=12)			
**Occupation**												
	Physicians	8 (20.5)		20 (38.4)		1 (9.1)		1 (8.3)		81 (71.1)		
	Nurses	1 (2.5)		1 (1.9)		1 (9.1)		0 (0)		8 (7)		
	Allied health personnel	4 (10.2)		3 (5.8)		1 (9.1)		1 (8.3)		25 (21.9)		
**Employer**												
	Work in privately funded health care	10 (25.4)		10 (19.2)		1 (9.1)		1 (8.3)		61 (53.5)		
	Work in publicly funded health care	3 (7.7)		14 (26.9)		1 (9.1)		1 (8.3)		53 (46.5)		
**eHealth experience**												
	No previous experience with eHealth	4 (10.2)		7 (13.4)		0 (0)		0 (0)		26 (22.8)		
	2 or more years of experience with eHealth	6 (15.3)		19 (36.5)		2 (18.2)		1 (8.3)		88 (77.2)		
**Urban versus rural experience**												
	eHealth experience only in urban settings	1 (2.5)		10 (19.2)		1 (9.1)		0 (0)		28 (24.6)		
	eHealth experience only in rural settings	0 (0)		0 (0)		0 (0)		0 (0)		2 (1.8)		
	eHealth experience in both rural and urban settings	12 (31.6)		14 (26.9)		1 (9.1)		1 (8.3)		84 (73.6)		
**Employment experience**												
	Predominant eHealth experience in clinics	0 (0)		6 (11.5)		1 (9.1)		1 (8.3)		26 (22.7)		
	Predominant eHealth experience in education	1 (2.5)		8 (15.3)		0 (0)		1 (8.3)		23 (20.6)		
	Predominant eHealth experience in technology support	3 (7.7)		3 (5.8)		0 (0)		0 (0)		16 (13.7)		
	Predominant eHealth experience in training	0 (0)		5 (9.6)		0 (0)		0 (0)		12 (10.5)		
	Predominant eHealth experience in monitoring and evaluation	1 (2.5)		7 (13.4)		1 (9.1)		0 (0)		20 (18)		
	Predominant eHealth experience in administration	1 (2.5)		2 (3.8)		1 (9.1)		0 (0)		10 (8.6)		
	Predominant eHealth experience in planning	0 (0)		3 (5.8)		0 (0)		0 (0)		7 (5.9)		

### eHealth Implementation Model Results

**Figure 2:**
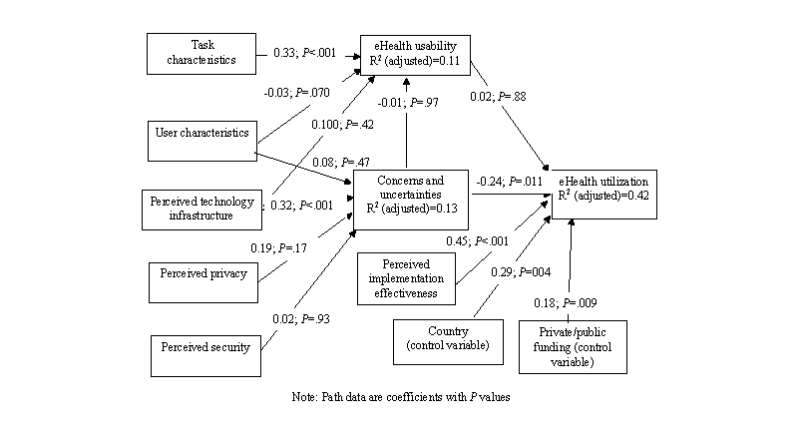
Model results for eHealth implementation issues.

Figure 2 shows the results calculated from the structural equation model, which was run with bootstrapping using 1000 subsamples. The calculated path coefficients for the proposed hypotheses, shown in Figure 2, are listed in Table 3. Hypotheses H1a, H2b, H3b, and H3c were supported, whereas the remaining hypotheses (H1b, H1c, H1d, H2a, H2c, H2d, and H3a) were not supported (all with *P*>.05).

Control variables (for the demographic categories in Table 2) were also run against the model, and those with significant results are included in Figure 2. These are (1) participant’s country and (2) employer private or public funded. The results for these control variables are shown in the lower part of Table 3.

The composite reliabilities and average variance extracted (AVE) for the reflective constructs are shown in Table 4. The composite reliability of a construct measures the reliability of the indicator variables included in the construct. All the composite reliabilities were above the accepted lower limit of 0.70 [26]. The AVE results measure the fit of the internal structure of the model. AVE is slightly below the accepted lower limit of 0.50 for *concerns and uncertainties*, but all the other values meet the lower limit within the rounding error; therefore, with this exception, the model has convergent validity. The heterotrait ratio of correlations [27], shown in Table 5, assesses the discriminant validity in the model. The resulting maximum value of 0.79 is below the 0.85 threshold, so discriminant validity is established. The adjusted R^2^ values for usability, concerns and uncertainties, and eHealth utilization are listed in Table 6.

Participants’ responses to the *eHealth utilization* construct were analyzed according to the extent to which the participants indicated that eHealth was used in their organization. The results, stratified by country, are shown as absolute values and percentages in Table 7.

**Table 3:** Calculated path coefficients and significance.

Relationship			Path coefficient		*P* value
**Hypothesis**					
	H1a: Task characteristics→^a^eHealth usability	0.33		<.001	
	H1b: User characteristics→eHealth usability	−0.03		.87	
	H1c: Perceived technology infrastructure→eHealth usability	0.10		.42	
	H1d: Concerns and uncertainties about eHealth→eHealth usability	0.01		.97	
	H2a: User characteristics→concerns and uncertainties	−0.08		.47	
	H2b: Perceived technology infrastructure→concerns and uncertainties	−0.32		<.001	
	H2c: Perceived privacy→concerns and uncertainties	0.20		.17	
	H2d: Perceived security→concerns and uncertainties	0.02		.93	
	H3a: Usability→eHealth utilization	0.02		.88	
	H3b: Concerns and uncertainties→eHealth utilization	−0.24		.01	
	H3c: Perceived implementation effectiveness→eHealth utilization	0.45		<.001	
**Significant control variables**					
	Country of participant→eHealth utilization	0.29		.004	
	Private or public funding→eHealth utilization	0.18		.009	

^a^Arrows represent the directional relationships of the coefficients.

**Table 4:** Composite reliabilities and average variance extracted for reflective constructs.

Construct	Composite reliability	AVE^a^
Concerns and uncertainties	0.74	0.42
Perceived implementation effectiveness	0.89	0.73
Perceived privacy	0.88	0.71
Perceived security	0.72	0.49
Perceived technology infrastructure	0.86	0.68
Perceived usability	0.85	0.59
Task characteristics	0.78	0.48
User characteristics	0.89	0.72

^a^AVE: average variance extracted.

**Table 5:** Discriminant analysis via heterotrait-monotrait ratio of correlations.

Constructs	Concerns and uncertainty	Perceived implementation effective	Perceived privacy	Perceived security	Perceived technology infrastructure	Task characteristics	Usability	User characteristics
Perceived implementation effectiveness	0.36	—^a^	—	—	—	—	—	—
Perceived privacy	0.36	0.16	—	—	—	—	—	—
Perceived security	0.17	0.14	0.79	—	—	—	—	—
Perceived technology infrastructure	0.46	0.54	0.04	0.10	—	—	—	—
Task characteristics	0.33	0.77	0.25	0.23	0.57	—	—	—
Usability	0.19	0.32	0.27	0.24	0.25	0.48	—	—
User characteristics	0.25	0.53	0.14	0.10	0.44	0.48	0.15	—
eHealth utilization	0.35	0.53	0.02	0.18	0.44	0.55	0.33	0.24

^a^Not applicable.

**Table 6:** Adjusted R^2^ from model calculations.

Latent variable	Adjusted R^2^
Usability	0.12
Concerns and uncertainties	0.13
eHealth utilization	0.42

**Table 7:** Participants’ responses to the question “indicate to what extent eHealth is used in your organization.”

Extent of eHealth use in my organization	Total (N=114), n (%)	Egypt (n=52), n (%)	India (n=39), n (%)	Nigeria (n=12), n (%)	Kenya (n=11), n (%)
Never	3 (2.6)	3 (5.8)	0 (0)	0 (0)	0 (0)
To a very small extent	22 (19.3)	12 (23.1)	9 (23.1)	0 (0)	1 (9.1)
To a small extent	24 (21.1)	12 (23.1)	4 (10.2)	5 (41.6)	3 (27.3)
To a moderate extent	35 (30.7)	14 (26.9)	15 (38.5)	4 (33.3)	2 (18.2)
To a fairly great extent	17 (14.9)	8 (15.4)	6 (15.4)	2 (16.7)	1 (9.1)
To a great extent	6 (5.3)	3 (5.8)	2 (5.1)	0 (0)	1 (9.1)
To a very great extent	7 (6.1)	0 (0)	3 (7.7)	1 (8.3)	3 (27.3)

## Discussion

### Principal Findings

The focus of our study was on providers and support staff with applied experience in eHealth and the extent of use they perceived of eHealth in their organizations. The structural equation model we developed resulted in an estimate of the extent of eHealth utilization in their organizations (adjusted R^2^=0.42). This result is in the range of a moderately good result [28] for an exploratory study.

In the model, only task characteristics contributed significantly to usability (Figure 2). When the model was run without control variables in place, it gave an adjusted R^2^ of 0.310 for eHealth utilization, with the path coefficient usability→eHealth utilization significant at 0.18 (*P*=.01). Other path coefficients changed very little, but this one became nonsignificant at a value of 0.015 (*P*=.88) when the 2 significant control variables (country and public or private employment) were included in the final model. This result tells us that there was variability among the opinions of the participants about eHealth utilization that depended upon their home country and their employer (private or public).

### eHealth Utilization

eHealth utilization in participant organizations was dealt with in question 10 of Multimedia Appendix 1. Its raw data results were analyzed and are presented in Table 7. These results show a similar distribution of engagement level of eHealth in health care organizations in each of the 4 countries. The median use of eHealth by the LRC participant organizations was *to a moderate extent,* as indicated by calculations from the raw data in response to the question in Multimedia Appendix 1 *the extent of eHealth use in your organization*. This suggests a generally favorable reaction to the applicability of eHealth to health care organization work. Although the sample sizes of the Nigerian and Kenyan responses were too small for statistical comparisons, there was little difference in their average results from the Egyptian and Indian responses.

### Privacy and Security

Privacy and its related supporting functionality, that is, security, were not found to be significant to the model construct eHealth concerns and uncertainties and, thus, to eHealth utilization, which is in agreement with similar studies [7,18]. Although this may be the case for eHealth users such as our participants, patients themselves in other LRC studies were found to be concerned about privacy violations through secondary or unauthorized access [19,29]. However, the lack of significance in our model did not mean that privacy and security were unimportant to the participants. From the raw data for question 4 in Multimedia Appendix 1, the overall result was a median value of 7 (you strongly agree) and a mean value of 6.3 (you agree) for the 3 positive statements in the questionnaire about the relevance of privacy, and similar results for the 3 positive statements in the questionnaire about the relevance of security (Multimedia Appendix 1). These are favorable results that did not have a significant impact on the model results because they did not seem to be of concern to most participants in relation to eHealth utilization.

### Relevance to Previous Literature

The recent history of eHealth implementation and experimentation in many LRCs has resulted in research and publication of many relevant results, including those that touched on sustainable implementation of eHealth in these countries [3,7,17,18,30,31]. In addition, Mauco et al [32] developed and validated an eHealth readiness assessment framework for developing countries. It includes a comprehensive set of readiness factors, including organizational, technological or infrastructural, government, societal, health care provider, engagement, core, and public- or patient-related. Another study by Ahmed et al [13], adapted from the study by Broens et al [12], synthesized 5 mobile health and telehealth (generalizable to eHealth) success factors, which we referenced in the introduction to this paper. Our research focused on 2 of these success factors (technology and its support infrastructure and user acceptance) to portray their effects on user perceptions.

The implementation of eHealth systems in LRCs differs from past activities with the more mature systems in developed nations. For example, many LRCs have been implementing pilot eHealth systems, some of which have been successful whereas others have not. Some of these implementations have ignored long-term effects, such as nonstandard systems that do not interoperate with other existing or planned systems [4]. Mistakes of this nature were also made when eHealth systems were initially being used in developed nations, and it is important to avoid making the same expensive mistakes in LRCs.

### Other eHealth Implementation Measures

Whether hospitals implement experimental or full eHealth applications, their operations are a source of data and user opinions that could be harvested to deduce predictions and possible causes of success or failure in future installations. The problem is that almost all related research on LRCs has been based on single installations or systematic reviews [3,7,22,23,32], and it is difficult to generalize from these to validate a theoretical framework. We note that there are organizations that publish hospital rankings in different regions and countries, including most, if not all, LRCs (eg, *Ranking Web of Hospitals* [33]). These rankings do not specifically include eHealth considerations. More specific to hospital implementations of eHealth is the Electronic Medical Record Adoption Model (EMRAM; HIMSS Analytics) [34]. EMRAM is an 8-level maturity model, beginning at level 0 (no eHealth facilities) and improving to level 7 (virtually complete implementation of eHealth, including electronic medical records, external digital links, privacy, security, disaster recovery, data analytics, and data governance). Forward-looking hospital managers aspire to move upward on this scale. Although few hospitals outside the United States have reached levels 6 or 7, many hospitals in the United States have reached levels 6 or 7. Although the EMRAM approach has been used primarily in developed nations, its advantage is that it encourages hospitals to modernize their facilities through eHealth implementation in a carefully managed manner. It also helps the hospital management to look for advice from other hospitals to help them move more effectively to a higher level of eHealth implementation. The need for this more organized approach is similar in LRCs, except that LRC hospitals are often starting at an introductory, low level of eHealth use, where initial eHealth system adoption requires extensive changes in hospital operations and employee training.

### Implications for Future Design

Our research takes the first step in the use of local eHealth experience by combining the opinions of actual eHealth end users from several LRCs. Regardless of the digital design of eHealth systems with which participants worked, the results of this study can be generalized to other proposed installations. Our findings could ultimately influence the design of eHealth systems, apps, and interfaces.

We also believe that we have demonstrated in a small way how to improve the general theory of eHealth implementations in LRCs by assessing a simultaneous combination of opinions of end users about 2 of the main eHealth success factors [13]. By redesigning the model; extending the survey scope; and expanding the participant audience to users, planners, developers, decision makers, and politicians, an evaluation of all 5 success factors [13] in multiple eHealth installations could be undertaken. This would help LRC planners, aided by a modified maturity model approach, to develop an appreciation of the impact of the various factors [35] that may differ among LRCs or among different installations in the same LRC, through the expressed opinions of participants. This would also help to direct strategic investments in eHealth more effectively.

### Strengths and Limitations

Our eHealth research in LRCs was the first time this approach was used to gather users’ perceptions of how eHealth utilization differed among the countries involved in the study. Acknowledging some limitations in our survey design, it is clear that studies of this nature with revised survey formats could be undertaken on an expanded scale, involving participants with a wider range of eHealth backgrounds. The ultimate gain would be a wider development and understanding of an approach similar to maturity modeling to help the hospital management move ahead with eHealth implementation in an organized and optimal manner.

We received many interesting and useful comments from participants that we were unable to analyze and include here because of space limitations.

Limitations included our use of convenience sampling to identify participants, which was not fully representative of each country’s health care workforce. The participant response rate was also considerably less than statistically desirable, and we could have achieved a higher response rate if we had been able to pay participants a nominal fee.

## Appendix

Multimedia Appendix 1 Survey questionnaire.

Multimedia Appendix 2 Information, consent, and invitation to participate in the online survey.

Multimedia Appendix 3 Details of the survey process.

## Abbreviations

AVE: average variance extracted
EMRAM: Electronic Medical Record Adoption Model
LRC: low-resource country
