# Supplementary material for: eHealth Implementation Issues in Low-Resource Countries: Model, Survey, and Analysis of User Experience
Source: J Med Internet Res. 2021 Jun 18;23(6):e23715. doi: 10.2196/23715 (PMC8277330; doi:10.2196/23715)
Supplement: Multimedia Appendix 1 [file jmir_v23i6e23715_app1.docx]

**EHEALTH IMPLEMENTATION ISSUES IN LOW RESOURCE COUNTRIES:**

**A SURVEY OF RELEVANCE, NEED, AND USE OF EHEALTH IN YOUR COUNTRY**

**Definitions:** In this survey, you will need to know the following:

1. eHealth – a healthcare field that may involve one or more of medical informatics, public health and business, and refers to health services and information delivered or enhanced through the Internet and related technologies.
2. ICT – an abbreviation for Information and Communications Technology (which is essential for the implementation and support of eHealth).
3. **Personal Information**

1.1 The country I represent for this survey and where I currently live is (check one):

___ India

___ Jamaica

___ Kenya

___ Nigeria

- 1. I am primarily employed as a (check one):

___ Physician

___ Nurse

___ Other (please describe here) ____________________________

- 1. My healthcare work is mainly funded (check one):

___ Privately

___ Publicly

- 1. My years of post-secondary education is: (check one)

___0

___1

___2

___3

___ more than 3

- 1. My years of experience with eHealth are approximately (check one):

___ 0

___ 1

___ 2

___ 3

___ more than 3

- 1. The percentages of my eHealth experiences have been in the following environments (total of percentages must be 100%).

___ % Urban

___ % Rural

- 1. My experience with eHealth has been in the following (check those that apply, using 1 for your most predominant eHealth experience, and so on to 5 as the least amount of experience). Leave all others blank:

___ clinical

___ technical support

___ training

___ education

___ administration

___ planning

___ monitoring and evaluation

___ other (please indicate) _______________________________________

Please click on a number from 1 to 7 to show your level of agreement with the statement for your country, pertaining ONLY to whether your work is financed publicly or privately, (as you indicated above in question 1.3).

Click **1** if you Strongly Disagree, **2** if you Disagree, **3** if you Disagree Slightly, **4** if you neither Agree nor Disagree, **5** if you Agree Slightly, **6** if you Agree, **7** if you Strongly Agree, and **DK** if you don’t know.

| 1. ***Technology Infrastructure*** |  |
| --- | --- |
| 2.1 Our ICT infrastructure to support eHealth is available 24/7 | 1 2 3 4 5 6 7 DK |
| 2.2 There are skilled human resources to implement and support ICT infrastructure. | 1 2 3 4 5 6 7 DK |
| 2.3 The ICT infrastructure, including networking, is good quality | 1 2 3 4 5 6 7 DK |
|  |  |
| 1. ***eHealth Usability*** |  |
| 3.1 eHealth technology that has been adopted is easy to learn | 1 2 3 4 5 6 7 DK |
| 3.2 eHealth technology that has been adopted is easy to use | 1 2 3 4 5 6 7 DK |
| 3.3 eHealth technology that has been adopted is flexible | 1 2 3 4 5 6 7 DK |
| 3.4 eHealth technology that has been adopted is useful | 1 2 3 4 5 6 7 DK |
|  |  |
| 1. ***Privacy*** |  |
| 4.1 Safeguarding anonymity is highly important for eHealth data | 1 2 3 4 5 6 7 DK |
| 4.2 Confidentiality of medical results is highly important for eHealth systems | 1 2 3 4 5 6 7 DK |
| 4.3 Protection from outsider access is highly important for eHealth data | 1 2 3 4 5 6 7 DK |
|  |  |
| 1. ***Security*** | 🡨1=Strongly Disagree  4=Neutral  7=Strongly Agree 🡪 |
| 5.1 The highest possible data security protection is of high importance for eHealth devices | 1 2 3 4 5 6 7 DK |
| 5.2 Determination of where and how eHealth data should be stored or transferred is of high importance | 1 2 3 4 5 6 7 DK |
| 5.3 Strict access control for eHealth data is of high importance | 1 2 3 4 5 6 7 DK |
|  |  |
| 1. ***eHealth Implementation Effectiveness*** |  |
| 6.1 In our organization, eHealth was effectively implemented. | 1 2 3 4 5 6 7 DK |
| 6.2 Our effort to integrate eHealth in our organization was acceptable. | 1 2 3 4 5 6 7 DK |
| 6.3 The implementation of eHealth in our organization is considered to be a success. | 1 2 3 4 5 6 7 DK |
|  |  |
| 1. ***Concerns and Uncertainties About eHealth*** |  |
| 7.1 There are funding limitations that may prevent long term implementations of eHealth | 1 2 3 4 5 6 7 DK |
| 7.2 There is a lack of infrastructure (electricity, network and Internet access) available to support eHealth systems | 1 2 3 4 5 6 7 DK |
| 7.3 A lack of standards adoption prevents scaling-up of successful eHealth implementations | 1 2 3 4 5 6 7 DK |
| 7.4 Acceptance of eHealth technology by the community may be limited by social factors (e.g., socio-economic, cultural or geographic barriers and quality of care) | 1 2 3 4 5 6 7 DK |
|  |  |
| 1. ***Individual Characteristics*** |  |
| 8.1 In my organization, eHealth users are knowledgeable when it comes to utilizing such technology | 1 2 3 4 5 6 7 DK |
| 8.2 In my organization, eHealth users possess a high degree of expertise | 1 2 3 4 5 6 7 DK |
| 8.3 In my organization, eHealth users are skilled at using the technology | 1 2 3 4 5 6 7 DK |
|  |  |
| 1. ***Task Characteristics*** |  |
| 9.1 In my organization, the eHealth system is used to look up treatment options for health conditions | 1 2 3 4 5 6 7 DK |
| 9.2 In my organization, the eHealth system is used to record and access patient data in medical records | 1 2 3 4 5 6 7 DK |
| 9.3 In my organization, the eHealth system is used to communicate and consult with healthcare providers | 1 2 3 4 5 6 7 DK |
| 9.4 In my organization, the eHealth system is used to gather and evaluate medical evidence to inform a decision | 1 2 3 4 5 6 7 DK |
|  |  |
| ***10. eHealth Utilization*** |  |
| 10.1 Please indicate to what extent eHealth is used in your organization (check one only). |  |
| Never |  |
| To a very small extent |  |
| To a small extent |  |
| To a moderate extent |  |
| To a fairly great extent |  |
| To a great extent |  |
| To a very great extent |  |

| 1. You do not need to complete the following if you do not want to, but if you do, please enter any comments that you have about eHealth that you believe have not been covered adequately by any previous items. In particular: |
| --- |
| - 1. List three or more reasons for wanting more efforts to implement eHealth in your country |
| - 1. List three or more reasons for not wanting more efforts to implement eHealth in your country |
| - 1. Please feel free to enter any additional comments you have about eHealth in your region and in your country: how it is being implemented, what is lacking in this service, and so on. |

Thank you so much for your assistance in this matter. Your input about eHealth in your society will help us in understanding ways in which eHealth might be implemented in some way that will bring better healthcare to your country in a sustainable manner.

**For all survey participants**

Please press the <submit> button to submit your survey. You will no longer be able to make any revisions to your survey information.

If you DO NOT WISH to participate in the draw for a $50 US prize among survey participants from your country, press the <exit> button which will end your online participation.

If you DO WISH to participate in the random draw for a $50 US prize among survey participants from your country, please complete the following. This information will be stored separately from your survey submission so it will not be possible to relate your identity to the survey you have submitted. It will be used to contact you if you are randomly selected for the prize.

The country I represent for this survey and where I currently live (check only one):

___ India

___ Jamaica

___ Kenya

___ Nigeria

My name ________________________________________

A telephone number where I can be reached_____________

Please press the <exit> button which will submit your identification and end your participation in the study.
