# Supplementary material for: eHealth Implementation Issues in Low-Resource Countries: Model, Survey, and Analysis of User Experience
Source: J Med Internet Res. 2021 Jun 18;23(6):e23715. doi: 10.2196/23715 (PMC8277330; doi:10.2196/23715)
Supplement: Multimedia Appendix 2 [file jmir_v23i6e23715_app2.docx]

**Barriers to eHealth in Low Resource Countries:**

**Information, Consent and Invitation to Participate in Research**

This research program originated and is being coordinated by researchers at McMaster University in Canada. These researchers have a long-standing interest in eHealth and its potential for improvements in healthcare in low resource countries. The team is led by Dr. Norm Archer ([archer@mcmaster.ca](mailto:archer@mcmaster.ca)) and Dr. Maryam Ghasemaghaei ([ghasemm@mcmaster.ca](mailto:ghasemm@mcmaster.ca)). Other researchers involved with the project include Adekunle Ajiboye, Dr. Ann McKibbon, Dr. Deborah DiLiberto, and Dr. Cynthia Lokker.

Your Country Coordinator is ___________..

**Introduction**

- Before agreeing to take part in this study, please read the information in this research consent form. It includes details you need to know in order to decide if you wish to take part in the study.
- All research is voluntary; if you have any questions, please contact your country coordinator or a principal investigator (Dr. Norm Archer) at any time. You should not agree to participate in this study until you understand the information.

**Background**

eHealth is the use of information and communications technologies (ICT) in support of healthcare disciplines, including health surveillance, healthcare services, and health education, innovation and research. eHealth has the potential to improve preventive care and treatment delivery for many types of illness to many people in developing countries, greatly improving health service efficiency while improving their health outcomes.

**Purpose of the Research**

The purpose of this research is to develop and validate a model of the factors that influence the adoption of eHealth in low resource countries, based on the challenges to eHealth adoption that have been identified through a thorough review.

**If you agree to participate in this study:**

Your opinions and those of other participants in this survey who have worked with eHealth in your country will help us evaluate barriers to the sustainable use of eHealth. This in turn may assist your governments and healthcare agencies in improving healthcare in your country.

**Procedures you will be asked to carry out, as part of the study:**

You are asked to complete a survey where you respond to a number of statements concerning your opinions about eHealth and its use in your country. You are then asked to add any further comments you have about eHealth that you feel were not covered by the statements.

**Potential Harms (Injury, Discomforts, or Inconvenience)**

- There are no known potential harms to participants in this study
- If you are distressed in any way not currently known to the researchers, you will be able to terminate your work on the survey without any penalty, except that you will no longer qualify to enter the random prize draw to be won by a person who completes the survey.

**Potential Benefits**

- The intent of this study is to determine if there may be additional solutions to implementing eHealth in your country that might be beneficial to your healthcare system.

**Protecting Your Information**

- Any information you enter during the survey will be kept confidential, and only the investigators will have access to the data.
- Data will be aggregated for the final report in such a manner that no person entering data for the survey will be identifiable from the study results.
- Following completion of the survey, all data collected will be stored on a computer with password security access at McMaster University for a period of three years. The data will then be destroyed.

**Study Results**

The study results will be available online when the research has been completed, and you will have access to a published document or documents that present the results.

**Potential Time to Participate, and Reimbursement to the Participant**

- The total time for you to complete the survey is expected to average about 7 minutes.

You will not be reimbursed for your time taken to complete the survey, but you will be entered in a random draw for a prize to be awarded to a participant from your country in this research project.

**Participation and Withdrawal**

- Participation in this research study is voluntary, and no record will be kept of your participation unless you choose to enter the random draw. Once the random draw is completed, all records of your participation will be erased.
- If you decide to participate in this study you can change your mind without giving a reason, and you may withdraw during your participation at any time without any effect.

**New Findings or Information**

- We may learn new things about the implementation of eHealth in low resource countries during this study. If so, these will be included in the final report resulting from the analysis of the data.

**Questions About the Study**

If you have questions or need more information about the survey process, please contact Dr. Norm Archer at: [archer@mcmaster.ca](mailto:archer@mcmaster.ca) , +1-905-525-9140 Ext. 23944.

This study has been reviewed by the McMaster University Research Ethics Board and received ethics clearance. If you have concerns or questions about your rights as a participant or about the way the study is conducted, please contact:

McMaster Research Ethics Secretariat

Telephone: +1 (905) 525-9140 ext. 23142

C/o Research Office for Administrative Development and Support

E-mail: [ethicsoffice@mcmaster.ca](mailto:ethicsoffice@mcmaster.ca)

If you agree to participate in the survey, please click on the following link. Your agreement to participate in the survey is implied when you click on the link.

<Online Link to McMaster University Survey>
