# Supplementary material for: eHealth Implementation Issues in Low-Resource Countries: Model, Survey, and Analysis of User Experience
Source: J Med Internet Res. 2021 Jun 18;23(6):e23715. doi: 10.2196/23715 (PMC8277330; doi:10.2196/23715)
Supplement: Multimedia Appendix 3 [file jmir_v23i6e23715_app3.docx]

Checklist for Reporting Results of Internet E-Surveys (CHERRIES)

(A Paper Format of the Questionnaire Used is in Multi-Media Appendix 1)

| Item Category |  | Checklist Item |
| --- | --- | --- |
| Design | Survey Design | Target Population: eHealth end-users in Low Resource Countries. This was a convenience sample Sampling Frame: eHealth end-users known to Country Coordinators (CCs) in India, Nigeria, Egypt and Kenya. |
| IRB Approval | Approval | Approved by McMaster University Research Ethics Board, who also checked for approval with a representative from each of the four target countries |
|  | Informed Consent | See attached form (Pages 4-6) |
|  | Data Protection | Surveys were developed on Qualtrics System software and accessed via the Internet. Only the researchers had access to the survey data. Participants accessed the survey site by invitation only through a password provided to them. |
| Development and Pre-Testing | Development & Testing | The survey was developed from a questionnaire approved by the researchers. It was pre-tested by researchers for authenticity and usability. |
| Recruitment Process & Sample Description | Closed Survey | This was a closed survey which participants accessed by invitation only from the CC for the country where the participant resided. |
|  | Contact Mode | Initial contacts were made by CCs via telephone or Internet (e-mail). They contacted participants known in advance to have eHealth experience. |
|  | Advertising | None. Participants for each country were recruited by the CC for that country. |
| Survey Administration | Web/E-mail | Posted on a web site, with authorized access only |
|  | Context | Not open to the public |
|  | Mandatory/Voluntary | Accessed by eHealth users who agreed with the CCs that they would participate |
|  | Incentives | A random draw prize of $50 US was offered by the CC in each country for those wanting to participate in the draw. |
|  | Time Frame | March 15 2019 to October 15 2019 |
|  | Randomization of Questionnaire Items | No |
|  | Adaptive Questioning | Not used |
|  | Number of Items per Page | 3 |
|  | Number of Screens | 18 |
|  | Completeness Check | Three dry runs were used to check for questionnaire completeness. Non-responses were an option for each item (for the main questionnaire, instead of clicking on a Likert scale number from 1 to 7, participants could click on DK (Don’t Know)) |
|  | Review Step | Participants could go back to previous answers through a back button, and could also take a break, with a total completion time limitation of 7 days. |
| Response Rates | Unique Site Visitors (Invitations to Participate) | 177 |
|  | View Rate | 177/177 = 100% |
|  | Participation Rate | 136/177 = 77% |
|  | Completion Rate | 117/177 = 66% |
| Preventing Multiple Entries from the Same Individual | Cookies Used | Participation was allowed for invited users only. Unique user identifiers were used, with individual codes reserved that were based on the e-mail address of each participant. Each identifier could be used only once, to prevent duplicate entries from the same participant. |
|  | Log File Address | Analysis was not necessary since individuals were prevented from participating more than once |
|  | Registration | Registration was managed through participant details received from Country Coordinators who recruited potential participants |
| Analysis | Handling Incomplete Questionnaires | Questionnaires from participants who did not complete the questionnaire were not used |
|  | Questionnaires Submitted with an Atypical Time Stamp | Participants were limited to a maximum time of 7 days to complete data entry; they could start and stop data entry as they needed to, since these were people subject to interruptions from their clientele. |
|  | Statistical Correction | Entries with a continuous and large number of similar Likert scale responses over multiple constructs were dropped from the study (there were only 3 of these). Missing data for each Likert response were replaced by the mean of the overall level for that response. |
